# Association between right heart catheterization hemodynamics and glycosylated hemoglobin levels in adults with heart failure with reduced ejection fraction

**DOI:** 10.1097/XCE.0000000000000285

**Published:** 2023-06-21

**Authors:** Gaspar Del Rio-Pertuz, Cristina Morataya, Kanak Parmar, Zeyad Elharabi, Daniel Davis, Mostafa Abohelwa, Ozman Ochoa, Alison Tran, Kenneth Nugent, David Paniagua, Erwin Argueta-Sosa

**Affiliations:** a Department of Internal Medicine, Texas Tech University Health Sciences Center; b Division of Pulmonary and Critical Care Medicine, Department of Internal Medicine, Texas Tech University Health Sciences Center Lubbock; c Department of Internal Medicine, Baylor College of Medicine, Houston; d Division of Cardiology, Department of Internal Medicine, Texas Tech University Health Sciences Center, Lubbock, Texas, USA

**Keywords:** HbA1c, hemodynamics, HFrEF, right heart catheterization

## Abstract

This study hypothesized that elevated glycosylated hemoglobin (HbA1c) levels are associated with abnormal right heart catheterization (RHC) hemodynamic parameters in patients with heart failure with reduced ejection fraction (HFrEF) and no prior diagnosis of diabetes.

**Methods:**

Retrospective cohort study of adult patients with HFrEF and no prior diagnosis of diabetes who underwent RHC and had HbA1c levels measured 30 days before or after the RHC. This study excluded patients who had received blood transfusions within 90 days prior to HbA1c measurement and patients with known diabetes. Univariate and multivariate regression analyses adjusted for age, sex, and BMI were used to test for an association between RHC hemodynamic parameters and HbA1c levels.

**Results:**

A total of 136 patients were included with a mean age of 55 ± 15 years and mean HbA1c was 5.99 ± 0.64%. Unadjusted univariate models showed that HbA1c is significantly associated with cardiac index (CI) by the Fick method and thermodilution, right atrial pressure (RAP), and mean pulmonary arterial pressure (MPAP). After multivariate analysis, for every one unit increase in HbA1c, there was a 0.19 and 0.26 L/min/m^2^ decrease in expected CI by thermodilution and by the Fick method (*P* = 0.03 and *P* < 0.01), respectively. For every one unit increase in HbA1c, there was a 2.39 mmHg increase in expected RAP (*P* = 0.01).

**Conclusion:**

Elevated HbA1c levels measured within 30 days before or after the index RHC in patients with a left ventricular ejection fraction <40% were associated with congestive hemodynamic parameters.

## Background

Diabetes mellitus (DM) and heart failure (HF) are frequently associated with clinical disorders. DM has a prevalence of approximately 25% in stable HF, compared to only 7% in the general population [1]. This relationship may be due in part to pathophysiological processes that underlie both HF and DM, including similar patterns of neurohormonal activation, endothelial dysfunction, and oxidative stress [2].

Right heart catheterization (RHC) is an invasive procedure that allows direct right heart pressure measurements and cardiac output (CO) estimation, with the goal of either assess hemodynamic status or diagnose certain cardiopulmonary diseases that are not identified with noninvasive tests. Although an RHC can help determine the patient’s volume status and distinguish HF from other disorders that cause circulatory instability, there is no established role for periodic invasive hemodynamic measurements in HF management [3].

HF may increase the propensity to develop insulin resistance with a subsequent increase in plasma glucose. Furthermore, it has been shown that patients with HF are more insulin resistant than normal controls [4–6]. This insulin resistance may lead to impaired peripheral vasodilation and increased left ventricular afterload causing hemodynamic derangements affecting cardiac performance. This could cause exercise intolerance as shown by a shorter 6-minute walk distance in patients with glucose intolerance compared to patients with normal glucose control [7].

Many landmark clinical trials have addressed the relationship between tight glycemic control and cardiovascular endpoints. However, the relationship between hemodynamics and glycemia has not been studied. Glycosylated hemoglobin (HbA1c) is the result of nonenzymatic hemoglobin glycosylation and indicates the average blood glucose concentration over the past 6–8 weeks [8]; this makes it a useful and simple index for predicting insulin resistance and dysglycemia. In addition, elevated glycemic levels have been associated with adverse outcomes in patients with HF [9]. Therefore, this study hypothesized that elevated HbA1c levels are associated with abnormal RHC hemodynamics in patients with HF with reduced ejection fraction (HFrEF) and no prior DM diagnosis.

## Methods

This study is based on a retrospective cohort of patients managed in the Internal Medicine clinics at Texas Tech University Health Sciences Center and University Medical Center in Lubbock, Texas, under IRB L21-159. It included all adult patients (>20 years) with HFrEF who underwent an RHC between 1 June 2015 and 1 June 2021 and had HbA1c levels measured within 30 days before or after the index catheterization. Included patients had to have a documented left ventricular ejection fraction of <40% by echocardiography 1 month after or up to 6 months before the index RHC was done. Patients were excluded if they had received blood transfusions within 90 days prior to HbA1c measurement or had a prior DM diagnosis, a history of hypoglycemic medication use, end-stage renal disease, and/or chronic corticosteroid use since these factors could affect HbA1c levels.

Hemodynamics were measured by RHC and dichotomized into normal and abnormal values based on conventional levels of clinical significance. Hemodynamic abnormalities were defined by a right atrial pressure (RAP) > 6 mmHg, right ventricular systolic pressure (RVSP) > 25 mmHg, right ventricular diastolic pressure > 8 mmHg, pulmonary artery systolic pressure (PASP) > 25 mmHg, pulmonary artery diastolic pressure > 15 mmHg, mean pulmonary artery pressure (MPAP) > 19 mmHg, and pulmonary artery wedge pressure > 15 mmHg. Using the Fick formula and thermodilution, CO < 4L/min and a cardiac index (CI) < 2.2 L/min/m^2^ were defined as abnormal.

Hemodynamic measurements were obtained with a Swan-Ganz pulmonary artery catheter (Edwards Lifesciences, Irvine, California, USA), a fluid-filled catheter. This catheter is connected to pressure transducers which have an accuracy, per factory specifications, from −3 mmHg to 50 mmHg. In this pressure range the minimum reading is −1 mmHg or −1%, and the maximum reading is +1 mmHg or +1% of current readings.

Numerical variables are reported with means and SD, and categorical variables are reported using counts and frequencies. Descriptive statistics were used to determine differences in patients with normal and abnormal cutoff hemodynamic parameters. Categorical variables (gender and ethnicity) were compared using chi-square tests, and numerical variables (age and BMI) were compared using *t*-tests. Fisher’s exact test was used instead of the chi-square test when cells had expected frequencies below 5.

Univariate linear regression analyses were used to evaluate the relationship between HbA1c as the predictor variable and RHC hemodynamics as the outcome variable. Additional RHC hemodynamic predictors were selected after performing a stepwise selection technique for model analysis. Multivariate regression analyses were performed by adjusting for age, gender, and BMI; regression coefficients and 95% confidence intervals are reported. A *P*-value <0.05 was considered to be statistically significant for all the tests. All statistical analyses were done in SAS 9.4 version for Windows.

## Results

**Fig. 1:**
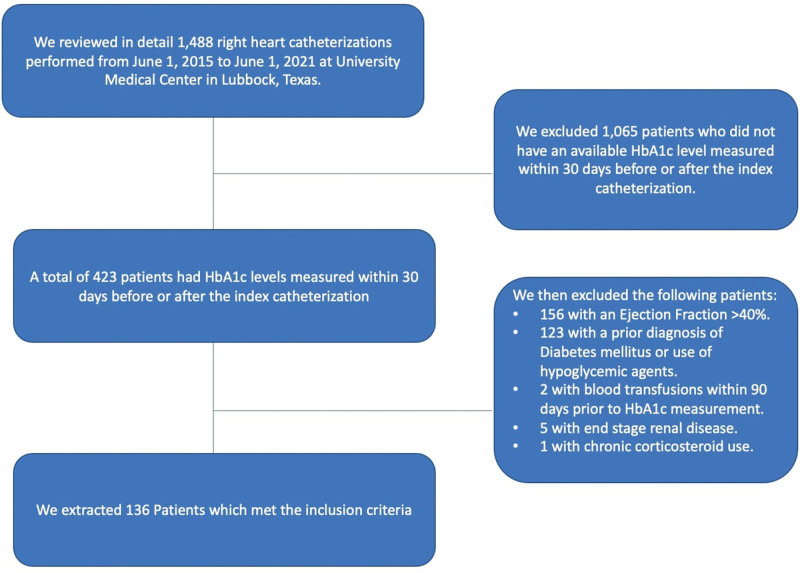
Consort diagram.

A total of 136 patients were included (Fig. 1); 102 patients (75%) were males, 68 patients (50%) were white, and 92 patients (68%) had non-ischemic cardiomyopathies (Table 1). The mean HbA1c was 5.99% ± 0.64%. There were no significant differences in age or gender between patients with normal and abnormal RHC hemodynamics. Ethnicity differed between normal and abnormal RVSP (Supplementary Material, Supplemental digital content 1, http://links.lww.com/CAEN/A41). Compared to patients with low RVSP pressures, patients with high RVSP pressures were more likely self-reported white ethnicity (53.91%, *P* = 0.009). The mean BMI differed in patients classified by CO measured by thermodilution, RAP, and RVSP. Compared to patients with abnormal parameters, patients with normal parameters had higher mean BMIs (Supplementary Material, Supplemental digital content 1, http://links.lww.com/CAEN/A41).

**Table 1:** Baseline clinical characteristics

Characteristics	Value
Demographics	
Age	54.53 (±14.96)
Gender (male)	102 (75.00%)
Race	
White	68/135 (50.37%)
Latino	33/135 (24.44%)
African American	11/135 (8.15%)
Other	23/135 (17.04%)
BMI	29.88 (±8.08)
Comorbidities	
Hypertension	93/135 (68.89%)
CAD	48/136 (35.29%)
COPD	17/136 (12.50%)
Asthma	8/136 (5.88%)
Cancer	8/136 (5.88%)
Chronic kidney disease	52/135 (38.52%)
GFR	64.42 (±11.30)
Hyperlipidemia	67/135 (49.63%)
Ischemic heart disease	44/136 (32.35%)
Medications	
Beta-blockers	89/135 (65.93%)
ACEI/ARB	67/133 (49.62%)
MR antagonists	25/129 (19.38%)
ARNI	18/121 (14.88%)
Laboratory	
HBA1C	5.99 (±0.64)
Serum creatinine	1.61 (±0.69)
Pro-BNP	10 763.41 (±12 021.52)
Troponin	164.23 (±637.05)
Echocardiography	
LVEF	24.84 (±6.33)
TAPSE	1.67 (±0.48)
Pulmonary artery systolic pressure	45.52 (±15.80)
Diastolic dysfunction	53/89 (59.55%)
Grade of diastolic dysfunction	
0	3/136 (2.21%)
1	10/136 (7.35%)
2	24/136 (17.65%)
3	12/136 (8.82%)
Right heart catheterization measurements	
Right atrial pressure	9.90 (±7.26)
Right ventricular systolic pressure	43.60 (±15.05)
Right ventricular diastolic pressure	4.68 (±7.44)
Pulmonary artery systolic pressure	45.52 (±15.80)
Pulmonary artery diastolic pressure	20.22 (±10.56)
Mean pulmonary artery pressure (MPAP)	29.54 (±11.29)
Pulmonary artery wedge pressure	19.66 (±10.01)
CO Fick	4.29 (±1.40)
CO thermo	4.38 (±1.48)
CI Fick	2.14 (±0.58)
CI thermo	2.19 (±0.62)

ACEI/ARB, angiotensin-converting enzyme inhibitors/angiotensin receptor blockers; ARNI, angiotensin receptor II blocker - neprilysin inhibitor; CAD, coronary artery disease; CI, cardiac index; CO, cardiac output; COPD, chronic obstructive pulmonary disease; GFR, glomerular filtration rate; HbA1c, glycosylated hemoglobin; LVEF, left ventricular ejection fraction; MR, mineralocorticoid receptor; Pro-BNP, prohormone B-type natriuretic peptide; TAPSE, tricuspid annular plane systolic excursion.

**Fig. 2:**
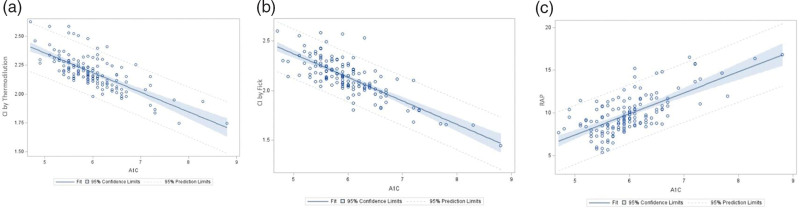
Fit plot of cardiac index by thermodilution (a), the Fick method (b), and right atrial pressure (c) adjusted by age, gender, BMI, and glycosylated hemoglobin levels.

Unadjusted univariate models showed that HbA1c is associated with CI measured by thermodilution (*P* = 0.04) or the Fick method (*P* < 0.01), MPAP (*P* = 0.04), and RAP (*P* = 0.01). After conducting multivariate analyses, for every one unit increase in HbA1c, there was a 0.19 and 0.26 L/min/m^2^ decrease in expected CI by thermodilution and by the Fick method, respectively (*P* = 0.03 and *P* < 0.01, Fig. 2a and b). For every one unit increase in HbA1c, there was a 2.39 mmHg increase in expected RAP (*P* = 0.01, Fig. 2c). There was no association between MPAP and HbA1c after adjustment (Table 2).

**Table 2:** Unadjusted univariate and adjusted multivariate linear regression analyses for right heart catheterization hemodynamics and HbA1c levels adjusted for age, gender, and BMI

	β	(95% CI)	*P*-value
Cardiac index by thermodilution			
Unadjusted			
HbA1c	−0.17	(−0.34 to−0.01)	**0.04**
Adjusted			
HbA1c	−0.19	(−0.36 to −0.02)	**0.03**
Age	−0.0003	(−0.01 to 0.03)	0.93
Gender (male)	0.03	(−0.22 to 0.28)	0.80
BMI	0.01	(−0.001 to 0.03)	0.07
Cardiac Index by Fick			
Unadjusted			
HbA1c	−0.23	(−0.38 to −0.08)	**0.003**
Adjusted			
HbA1c	−0.26	(−0.41 to −0.11)	**0.0009**
Age	0.006	(−0.001 to 0.01)	0.07
Gender (male)	−0.04	(−0.26 to 0.18)	0.71
BMI	0.013	(0.005 to 0.02)	**0.04**
Cardiac output by thermodilution			
Unadjusted			
HbA1c	−0.11	(−0.52 to 0.30)	0.59
Adjusted			
HbA1c	−0.22	(−0.59 to 0.15)	0.25
Age	−0.01	(−0.03 to 0.01)	0.21
Gender (male)	0.67	(0.12 to 1.23)	**0.01**
BMI	0.07	(0.04 to 0.10)	**<0.0001**
Cardiac output by Fick			
Unadjusted			
HbA1c	−0.29	(−0.66 to 0.08)	0.13
Adjusted			
HbA1c	−0.43	(−0.77 to −0.08)	**0.02**
Age	0.002	(−0.01 to 0.02)	0.76
Gender (male)	0.59	(0.08 to 1.10)	**0.02**
BMI	0.07	(0.04 to 0.10)	**<0.0001**
Mean pulmonary arterial pressure			
Unadjusted			
HbA1c	3.04	(0.09 to 5.98)	**0.04**
Adjusted			
HbA1c	2.89	(−0.11 to 5.89)	0.06
Age	0.03	(−0.10 to 0.16)	0.63
Gender (male)	−0.23	(−4.68 to 4.22)	0.92
BMI	0.08	(−0.16 to 0.33)	0.50
Pulmonary artery diastolic pressure			
Unadjusted			
HbA1c	1.34	(−1.44 to 4.13)	0.95
Adjusted			
HbA1c	1.13	(−1.70 to 3.95)	0.43
Age	−0.02	(−0.14 to 0.10)	0.76
Gender (male)	1.23	(−2.96 to 5.42)	0.56
BMI	0.13	(−0.10 to 0.36)	0.26
Pulmonary artery systolic pressure			
Unadjusted			
HbA1c	2.91	(−1.25 to 7.08)	0.17
Adjusted			
HbA1c	2.53	(−1.68 to 6.74)	0.24
Age	0.10	(−0.08 to 0.29)	0.27
Gender (male)	−0.11	(−6.35 to 6.14)	0.97
BMI	0.21	(−0.13 to 0.55)	0.23
Pulmonary artery wedge pressure			
Unadjusted			
HbA1c	2.36	(−0.27 to 4.99)	0.08
Adjusted			
HbA1c	2.28	(−0.39 to 4.94)	0.09
Age	0.02	(−0.10 to 0.14)	0.73
Gender (male)	−2.40	(−6.34 to 1.55)	0.23
BMI	0.08	(−0.15 to 0.30)	0.50
Right ventricular diastolic pressure			
Unadjusted			
HbA1c	−0.46	(−2.44 to 1.53)	0.65
Adjusted			
HbA1c	−0.40	(−2.42 to 1.63)	0.70
Age	−0.009	(−0.10 to 0.08)	0.84
Gender (male)	−1.09	(−4.13 to 1.94)	0.48
BMI	−0.03	(−0.19 to 0.14)	0.74
Right ventricular systolic pressure			
Unadjusted			
HbA1c	3.87	(−0.09 to 7.84)	0.06
Adjusted			
HbA1c	3.36	(−0.63 to 7.35)	0.10
Age	0.12	(−0.05 to 0.29)	0.16
Gender (male)	0.03	(−5.94 to 6.01)	0.99
BMI	0.27	(−0.06 to 0.60)	0.10
Right atrial pressure			
Unadjusted			
HbA1c	2.46	(0.57 to 4.34)	**0.01**
Adjusted			
HbA1c	2.39	(0.52 to 4.26)	**0.01**
Age	−0.06	(−0.14 to 0.02)	0.15
Gender (male)	−2.56	(−5.32 to 0.21)	0.07
BMI	0.08	(−0.07 to 0.24)	0.30

Bold values are statistically significant of *P* values.

CI, confidence interval; HbA1c, glycosylated hemoglobin.

## Discussion

In this retrospective cohort of patients with HFrEF, HbA1c levels were associated with RHC hemodynamic parameters. There was a positive correlation between HbA1c levels and RAP (*P* = 0.01) and a negative correlation between HbA1c levels and CI by both thermodilution and the Fick method (*P* = 0.03 and *P* < 0.01). These results correlate with congestive/abnormal hemodynamics (reduced CI and elevated RAP). Based on the current literature, this is the first study that has evaluated the association between RHC hemodynamics and glucose control in non-diabetic patients. This study agrees with prior results that evaluated the presence of impaired glucose and hyperinsulinemia in non-diabetic patients with HF [10]. However, the mechanism that interconnects HF with impaired glucose metabolism remains poorly understood [11].

**Fig. 3:**
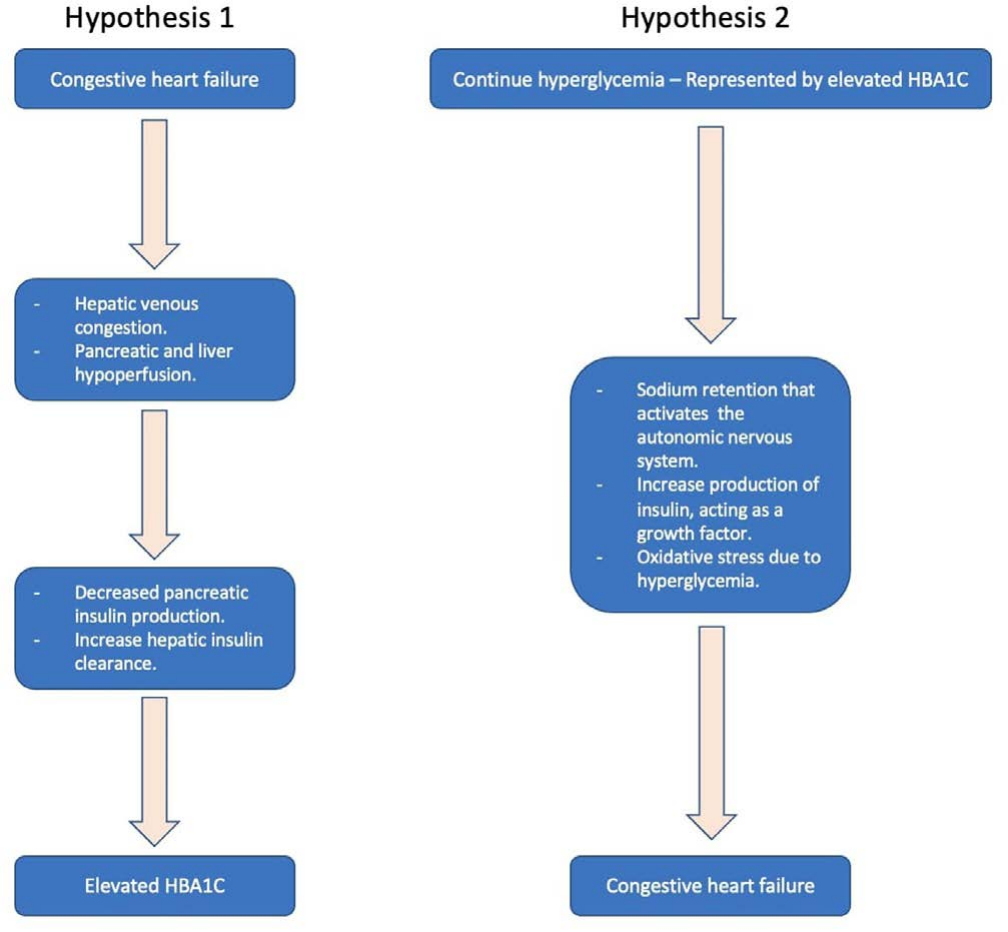
Central illustration describing the two hypotheses that might explain the association between congestive heart failure and elevated HbA1c.

Two main hypotheses might explain this association (Fig. 3). The first hypothesis is that HF causes glucose homeostasis dysregulation, a theory based on the tight physiologic relationship between the splanchnic circulation and the heart [12]. The liver’s vascular supply and high metabolic activity are highly vulnerable to circulatory disturbances. This occurs when decompensated HF causes passive hepatic venous congestion, which impairs liver function biochemically and causes histological changes [13]. These patients have increased bilirubin, aspartate aminotransferase, alanine aminotransferase, alkaline phosphatase, and gamma-glutamyl transferase levels and decreased albumin levels. In histological studies, congestion has been associated with bile canaliculi dilation and rupture, resulting in cirrhosis after long-standing damage [14]. This hepatic dysfunction, driven by right HF, might have a key role in serum glucose homeostasis by increasing hepatic insulin clearance contributing to impaired glucose control from decreasing systemic insulin levels [14]. Studies have shown that there are decreased systemic insulin levels and increased hepatic insulin clearance with increasing HF severity [11]. Liver function has an important effect on insulin levels since first-pass hepatic metabolism extracts 50% of secreted insulin [15]. Alternatively, prolonged decreases in splanchnic flow to the pancreas could create tissue damage and decrease the production of insulin resulting in poor glucose control [16].

The second hypothesis considers the effect of continuous hyperglycemia (represented by elevated HbA1c) on the vasculature leading to HF, which can be explained by several factors. First, hyperglycemia stimulates sodium retention that activates the autonomic nervous system causing hemodynamic derangements that lead to fluid overload causing HF [17,18]. Second, insulin might act as a growth factor and may contribute to myocardial dysfunction with increases in cardiac mass [18]. Third, hyperglycemia could damage the heart through oxidative stress, resulting in decreased cardiac contractility [19]. Fourth, HbA1c has an increased affinity for O_2_; therefore, higher HbA1c concentrations cause a disruption in oxygen release to cells and a reduced erythrocyte oxygen-carrying capacity leading to myocardial ischemia [20]. In patients with DM, a positive relationship between HbA1c and HF risk has been reported in several studies; the risk of hospitalization for HF increases 8–32% per 1% unit increase in HbA1c [1,21–23]. Matsushita *et al*. reported that impaired glucose metabolism even before DM development (HbA1c between 6.0 and 6.4%) is an independent risk factor for HF after adjustment for age, sex, race, and traditional cardiovascular risk factors [24]. Based on these previous findings, it is important to note that this study population had a mean HbA1c of 5.99% ± 0.64%.

The demographic information indicated that most patients were middle-aged white males with non-ischemic cardiomyopathies and BMIs in the overweight range. None of the patients included in the study had a BMI within normal limits. There are studies showing that BMI is positively correlated with HbA1c [25]. However, patients with congestive hemodynamics (reduced CI, and elevated RAP, RVSP, and MPAP) did not have a significantly higher BMI than patients without congestive RHC hemodynamics. This suggests that the elevated HbA1c in this population is not influenced by BMI.

This retrospective study has several limitations. Patients with possible confounders that could affect HbA1c levels in HF patients (patients with DM, hypoglycemic medication use, end-stage renal disease, chronic steroid use, and red blood cell transfusions within the past 3 months) were excluded from the study, but other possible confounders that were not excluded (i.e. anemia, diet, and lifestyle changes) could have influenced the results. This study suggests that abnormal hemodynamics in HFrEF could be associated with glycemic derangements, but this cannot be conclusive since in this study no causal effect experiments were performed. There are no experimental data available in the literature supporting this observation. After conducting multivariate analyses, HbA1c remained the primary predictor of RHC hemodynamics. However, results are limited to a specific model with preselected variables and do not include all the factors that could affect the HbA1c-RHC hemodynamic relationships. Considering these limitations, the findings of the present study should be interpreted as only hypothesis-generating and should not be overstated. More studies are needed to determine if an association between impaired glucose control and abnormal hemodynamics in HF is clinically relevant. If there is significant improvement in HbA1c after HF optimization, this simple test could potentially be used as a prognostic factor for disease severity and as a method to monitor fluid balance status and cardiac function over longer treatment periods, for example, over quarterly clinic visits.

### Conclusion

HbA1c levels measured within 30 days before or after the index RHC in patients with left ventricular ejection fractions <40% were associated with parameters that correlate with congestive hemodynamics (reduced CI and elevated RAP). A larger study is needed to determine if these associations are consistent in all of the hemodynamic parameters obtained during an RHC and to see if HbA1c levels reflect responses to treatment.

## Acknowledgements

### Conflicts of interest

There are no conflicts of interest.

## Supplementary Material


