# Supplementary material for: Association between right heart catheterization hemodynamics and glycosylated hemoglobin levels in adults with heart failure with reduced ejection fraction
Source: Cardiovasc Endocrinol Metab. 2023 Jun 21;12(3):e0285. doi: 10.1097/XCE.0000000000000285 (PMC10287124; doi:10.1097/XCE.0000000000000285)
Supplement: Supplementary file 1 [file xce-12-e0285-s001.pdf]

Supplementary Material

Supplementary Tables.

Table 1. Demographics by Fick Cardiac Index

		CI Fick <2.2 L/min/m² (83)	CI Fick ≥ 2.2 Lmin/m² (51)	*P* value
Age (years)		54.30	55.20	0.74
Gender (%)	Female	25.61	23.53	0.79
	Male	74.39	76.47	
Ethnicity (%)	White	54.32	45.10	0.64
	Latino	20.99	29.41	
	Afro-American	7.41	9.80	
	Other	17.28	15.69	
BMI (Kg/m²)		29.21	30.98	0.23

Table 2. Demographics by Cardiac Output Fick

		CO Fick <4L/min (59)	CO Fick ≥ 4 L/min (75)	*P* value
Age (years)		53.42	55.62	0.40
Gender (%)	Female	28.81	21.62	0.34
	Male	71.19	78.38	
Ethnicity (%)	White	53.45	48.65	0.92
	Latino	24.14	24.32	
	Afro-American	6.90	9.46	
	Other	15.52	17.57	
BMI (Kg/m2)		28.76	30.79	0.16

Table 3. Demographics by Cardiac Index Thermodilution

		CI Thermodilution <2.2 L/min/m² (64)	CI Thermodilution ≥ 2.2 Lmin/m2 (58)	*P* value
Age (years)		53.78	52.81	0.72
Gender (%)	Female	30.16	20.69	0.24
	Male	69.84	79.31	
Ethnicity (%)	White	51.61	46.55	0.38
	Latino	19.35	32.76	
	Afro-American	9.68	6.90	
	Other	19.35	13.79	
BMI (Kg/m2)		29.27	30.65	0.34

Table 4. Demographics by Cardiac Output Thermodilution

		CO Thermodilution <4L/min (54)	CO Thermodilution ≥ 4 L/min (68)	*P* value
Age (years)		54.87	52.10	0.31
Gender (%)	Female	33.96	19.12	0.06
	Male	66.04	80.88	
Ethnicity (%)	White	55.77	44.12	0.16
	Latino	17.31	32.35	
	Afro-American	5.77	10.29	
	Other	21.15	13.24	
BMI (Kg/m2)		27.91	31.50	**0.01**

Table 5. Demographics by Right Atrial Pressure.

		RAP ≤6 mmHg (49)	RAP >6 mmHg (86)	*P* value
Age (years)		57.76	52.67	0.06
Gender (%)	Female	16.33	30.59	0.07
	Male	83.67	69.41	
Ethnicity (%)	White	45.83	52.94	0.81
	Latino	29.17	22.35	
	Afro-American	8.33	7.06	
	Other	16.67	17.65	
BMI (Kg/m2)		27.73	30.94	**0.01**

Table 6. Demographics by Right Ventricular Systolic Pressure

		RVSP ≤ 25 mmHg (15)	RVSP >25 mmHg (117)	*P* value*
Age (years)		48.27	55.30	0.09
Gender (%)	Female	33.33	24.14	0.44
	Male	66.67	75.86	
Ethnicity (%)	White	20.00	53.91	**0.009**
	Latino	26.67	25.22	
	Afro-American	6.67	7.83	
	Other	46.67	13.04	
BMI (Kg/m2)		27.29	30.20	**0.02**

Table 7. Demographics by Right Ventricular Diastolic Pressure

		RVDP **≤ 8**mmHg (108)	RVDP >8 mmHg (24)	*P* value*
Age (years)		55.27	51.04	0.22
Gender (%)	Female	24.30	29.17	0.62
	Male	75.70	70.83	
Ethnicity (%)	White	49.06	54.17	0.86
	Latino	24.53	29.17	
	Afro-American	8.49	4.17	
	Other	17.92	12.50	
BMI (Kg/m2)		29.86	29.87	0.99

Table 8. Demographics by Pulmonary Artery Systolic Pressure

		PASP ≤ 25 mmHg (10)	PASP >25 mmHg (121)	*P* value*
Age (years)		51.18	54.75	0.45
Gender (%)	Female	27.27	25.00	1.00
	Male	72.73	75.00	
Ethnicity (%)	White	11.11	52.07	0.05
	Latino	44.44	24.79	
	Afro-American	0.00	8.26	
	Other	44.44	14.88	
BMI (Kg/m2)		27.67	30.10	0.34

Table 9. Demographics by Pulmonary Artery Diastolic Pressure

		PADP ≤ 15 mmHg (55)	PADP >15 mmHg (82)	*P* value
Age (years)		56.05	53.49	0.33
Gender (%)	Female	27.27	23.43	0.61
	Male	72.73	76.54	
Ethnicity (%)	White	44.44	54.32	0.73
	Latino	27.78	22.22	
	Afro-American	9.26	7.41	
	Other	18.52	16.05	
BMI (Kg/m2)		29.40	30.20	0.57

Table 10. Demographics by Mean Pulmonary Artery Pressure

		MPAP **≤19** mmHg (36)	MPAP >20 mmHg (101)	*P* value
Age (years)		55.11	54.32	0.79
Gender (%)	Female	27.78	24.00	0.65
	Male	72.22	76.00	
Ethnicity (%)	White	37.14	55.00	0.22
	Latino	25.71	24.00	
	Afro-American	11.43	7.00	

	Other	25.71	14.00	
BMI (Kg/m2)		27.94	30.57	0.09

Table 11. Demographics by Pulmonary Artery Wedge Pressure.

		PAWP ≤15 mmHg (51)	PAWP >15 mmHg (84)	*P* value
Age (years)		54.63	53.86	0.77
Gender (%)	Female	23.53	26.51	0.70
	Male	76.47	73.49	
Ethnicity (%)	White	52.00	48.19	0.76
	Latino	20.00	27.71	
	Afro-American	10.00	7.23	
	Other	18.00	16.87	
BMI (Kg/m2)		28.75	30.43	0.23

**P* value obtained from Exact Fisher's Test
